# Supplementary material for: Adaptation to criticality through organizational invariance in embodied agents
Source: Sci Rep. 2018 May 16;8:7723. doi: 10.1038/s41598-018-25925-4 (PMC5956029; doi:10.1038/s41598-018-25925-4)
Supplement: Supplementary file 1 — Supplementary Information [file 41598_2018_25925_MOESM1_ESM.pdf]

# Supplementary Information

## Adaptation to criticality through organizational invariance in embodied agents

Miguel Aguilera
Manuel G. Bedia

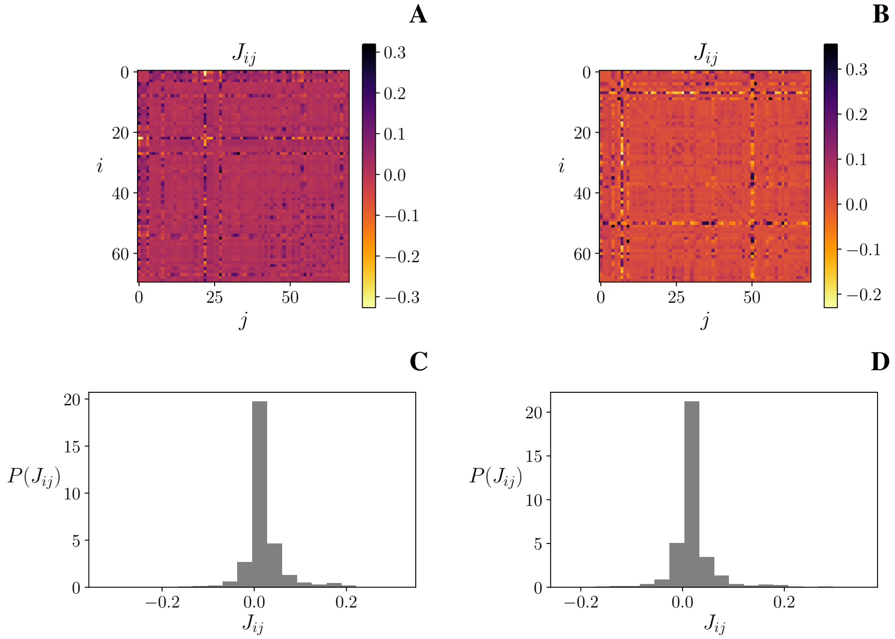


**Figure S1.** Example of the matrix of coupling values $J_{ij}$ for an agent with $N_h = 64$ hidden units for the Mountain Car **(A)** and Acrobot **(B)** embodiments. **(C-D)** Histogram of the probability distribution of the corresponding coupling matrix.

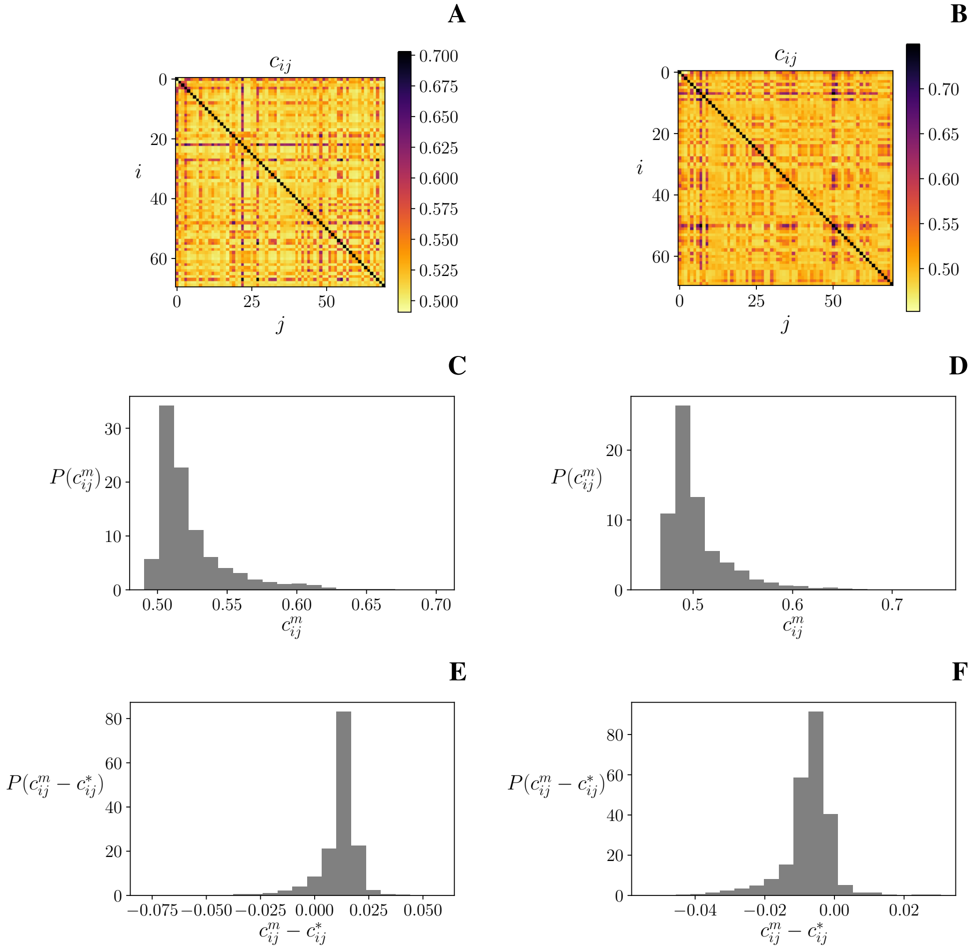


**Figure S2.** Example of the matrix of correlation values $c_{ij}^m$ for an agent with $N_h = 64$ hidden units for the Mountain Car **(A)** and Acrobot **(B)** embodiments during a simulation of $10^6$ simulation steps, reseting the agent's position and state every $5 \cdot 10^4$ simulation steps. **(C-D)** Histogram of the probability distribution of the corresponding correlation matrix. **(C-D)** Histogram of the probability distribution of the error between the correlations computed during simulation $c_{ij}^m$ and reference correlations assigned during training $c_{ij}^*$.
